# Synthetic Approaches and Clinical Application of Representative Small-Molecule Inhibitors of Cyclin-Dependent Kinase for Cancer Therapy

**DOI:** 10.3390/molecules29133029

**Published:** 2024-06-26

**Authors:** Ya-Tao Wang, Shi-Qi Jiang, Shao-Lin Zhang

**Affiliations:** 1 First People’s Hospital of Shangqiu, Shangqiu 476100, China; 2 Department of Anesthesiology, The First Hospital of China Medical University, Shenyang 110001, China; clarence6660010@163.com; 3 School of Pharmaceutical Sciences, Chongqing Key Laboratory of Natural Product Synthesis and Drug Research, Chongqing University, Chongqing 401331, China

**Keywords:** CDK, synthesis, application, small molecule, drugs

## Abstract

The regulation of the cancer cell cycle heavily relies on cyclin-dependent kinases (CDKs). Targeting CDKs has been identified as a promising approach for effective cancer therapy. In recent years, there has been significant attention paid towards developing small-molecule CDK inhibitors in the field of drug discovery. Notably, five such inhibitors have already received regulatory approval for the treatment of different cancers, including breast tumors, lung malignancies, and hematological malignancies. This review provides an overview of the synthetic routes used to produce 17 representative small-molecule CDK inhibitors that have obtained regulatory approval or are currently being evaluated through clinical trials. It also discusses their clinical applications for treating CDK-related diseases and explores the challenges and limitations associated with their use in a clinical setting, which will stimulate the further development of novel CDK inhibitors. By integrating therapeutic applications, synthetic methodologies, and mechanisms of action observed in various clinical trials involving these CDK inhibitors, this review facilitates a comprehensive understanding of the versatile roles and therapeutic potential offered by interventions targeting CDKs.

## 1. Introduction

One of the characteristic features of tumors is abnormal cell proliferation caused by cell division imbalance. Therefore, identifying and blocking the relevant targets of cell division is an important direction for tumor treatment. The division of eukaryotic cells follows a highly conserved process called the cell cycle. Each step of the cell cycle must occur in a sequential manner under the control of relevant signal checkpoints. CDKs control the transition from one stage of the cell cycle to the following stage. CDKs are activated when they interact with cyclins. Therefore, CDKs are potential targets for tumor treatment [1,2,3].

There are four phases in the cell cycle: G1 (the pre-deoxyribonucleic acid (DNA) synthesis phase), S (the DNA synthesis phase), G2 (the pre-cell division phase), and M (the cell division phase) [4]. So far, dozens of CDKs (CDK1/2/4/6/7/9, etc.) and cyclins (cyclin A/B/D/E/F/G/H, etc.) have been identified and play important roles in cell cycle regulation. For example, cyclin D-CDK4/6 initiates the cell cycle process, cyclin E-CDK2 regulates entry into the S phase, cyclin A-CDK2 regulates DNA replication in the S phase, and cyclin A/B-CDK1 triggers mitosis [5,6]. The development of CDK inhibitors as targeted anticancer agents has garnered significant interest within the academic community due to the link between the abnormal expression of most CDKs and the progression of cancer [7]. Over the past 20 years, dozens of CDK-targeting drugs have been studied in clinical trials, but only a few have been approved for clinical treatment. In the early stages of research, the discovered CDK inhibitors were mostly pan-CDK inhibitors or multi-CDK inhibitors. As they could effectively inhibit multiple CDKs or other kinases, they exhibited significant side effects, which hindered the entry of these two types of inhibitors into the market and clinical treatment. Therefore, in order to obtain CDK inhibitors that are secure, more efficient, and have reduced side effects for clinical treatment, researchers have conducted studies on selective CDK inhibitors. The US Food and Drug Administration (FDA) approved Palbociclib, the first selective CDK4/6 inhibitor, in 2015 for the purpose of treating breast cancer [8]. Subsequently, three selective CDK4/6 inhibitors, Ribociclib, Abemaciclib, and Trilaciclib, were also launched and used in clinical treatment [9,10,11]. In addition, the currently identified selective CDK inhibitors mainly target CDK1, CDK2, CDK4/6, CDK9, etc. [12]. CDK inhibitors have demonstrated their effectiveness in the treatment of various diseases, encompassing not only cancer but also non-cancer ailments such as central nervous system disorders, infectious diseases, and inflammatory diseases [13,14,15]. This highlights the significant role CDKs play in the development and progression of various cancers and non-cancerous ailments. 

**Figure 1:**
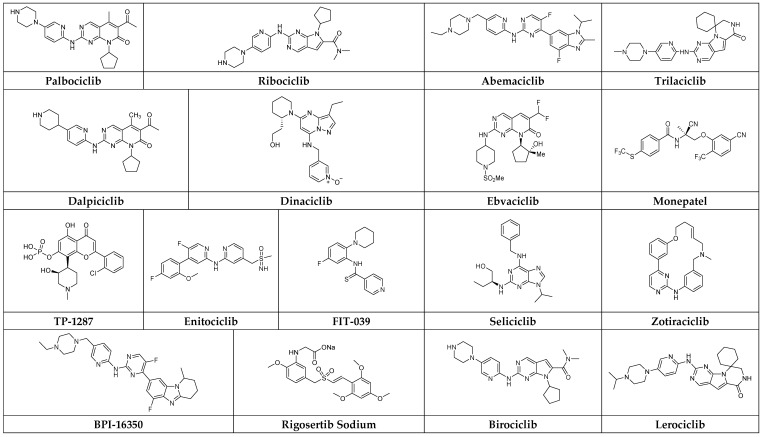
The chemical structures of representative CDK inhibitors in the clinic.

The advancements achieved in research on CDK inhibitors offer great potential for expanding treatment options in tumor therapy. A comprehensive examination of the complex synthetic methods used in developing CDK inhibitors at different stages (Figure 1), as well as their mechanisms of action in clinical settings, has the potential to accelerate the development of innovative pharmaceutical agents for tumor therapy.

## 2. Signaling Pathway of CDK

**Figure 2:**
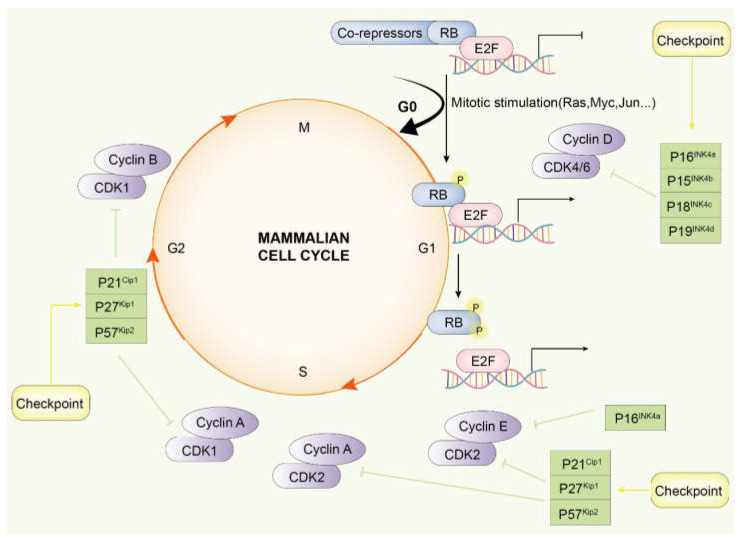
Signaling pathway of CDK.

The cell cycle progression and coordination of various cellular processes are significantly influenced by the CDK pathway [2]. CDKs, in partnership with cyclins, control the cell cycle by phosphorylating target proteins (Figure 2). They belong to a group of serine/threonine kinases. This pathway is crucial in guaranteeing smooth progressions through different stages of the cell cycle, such as the G1, S, G2, and M phases. Cyclins, regulatory proteins that undergo changes in abundance during the cell cycle, form the fundamental basis of the CDK pathway. Cyclins specifically bind to CDKs, stimulating their activation to initiate the phosphorylation of downstream substrates. These cyclin–CDK complexes tightly regulate progression through the cell cycle, ensuring the completion of each phase before advancing to the next. During the G1 phase, the initiation of the cell cycle heavily relies on the cyclin D-CDK4/6 complex. Its primary role is to phosphorylate the retinoblastoma protein (Rb), resulting in the liberation of the transcription factor E2F. This, in turn, triggers the activation of genes that are essential for DNA synthesis. The subsequent S phase involves DNA replication, orchestrated by cyclin E-CDK2. As cells proceed to the G2 phase, cyclin A-CDK2 takes over, coordinating preparation for mitosis. The transition to the M phase, or mitosis, is regulated by cyclin B-CDK1. This complex is responsible for initiating the breakdown of the nuclear envelope, chromatin condensation, and spindle formation. The proper execution of these events ensures the accurate segregation of chromosomes during cell division. The CDK pathway is tightly regulated by multiple mechanisms, including the presence of cyclin inhibitors, such as p16, p21, and p27, which act as checkpoints to halt cell cycle progression if necessary. Various diseases, including cancer, are associated with the dysregulation of the CDK pathway, where uncontrolled cell proliferation is a hallmark. In summary, the CDK pathway is a sophisticated and highly regulated network that orchestrates the progression of cells through the cell cycle. Its meticulous control is essential for maintaining genomic integrity and preventing aberrant cell proliferation. Understanding the intricacies of this pathway provides insights into both normal cellular processes and the development of therapeutic strategies for diseases involving cell cycle dysregulation.

## 3. Representative Small-Molecule CDK Inhibitors in the Clinic

### 3.1. Palbociclib

On 3 February 2015, the FDA in the United States granted accelerated approval to use Palbociclib for the treatment of advanced breast cancer in postmenopausal women who have an estrogen receptor (ER)+/human epidermal growth factor receptor 2-negative (HER2-) status [8]. This drug was subsequently commercialized in Chinese, Japanese, and European markets. Developed by Pfizer, Palbociclib, which is marketed under the trade name Vonjo, is the world’s first CDK 4/6 inhibitor (IC_50_ = 11 nM for CDK4, IC_50_ = 16 nM for CDK6) [16]. It functions by inhibiting the phosphorylation process of the retinoblastoma protein (Rb), effectively arresting cell cycle advancement from the G1 phase to S phase and impeding the proliferation of cancer cells. The potential for a substantial increase in progression-free survival (PFS) among patients with breast cancer is evident when Palbociclib and Letrozole are combined, instilling renewed hope and optimism [17]. This combination has demonstrated a significant increase in the duration during which the disease remains stable, offering a hopeful prospect for individuals impacted by breast cancer. Compared to injectable formulations, the capsule formulation of Palbociclib offers enhanced safety and improved patient adherence. Moreover, this medication does not induce gastrointestinal reactions that are commonly associated with traditional chemotherapy [18]. The commonly observed adverse effects of Palbociclib include a reduction in neutrophil and white blood cell counts, fatigue, a decreased red blood cell count, and nausea [8]. It is contraindicated in patients with severe hepatic impairment or individuals who have a known hypersensitivity to the medication or any of its constituents.

**Scheme 1:**
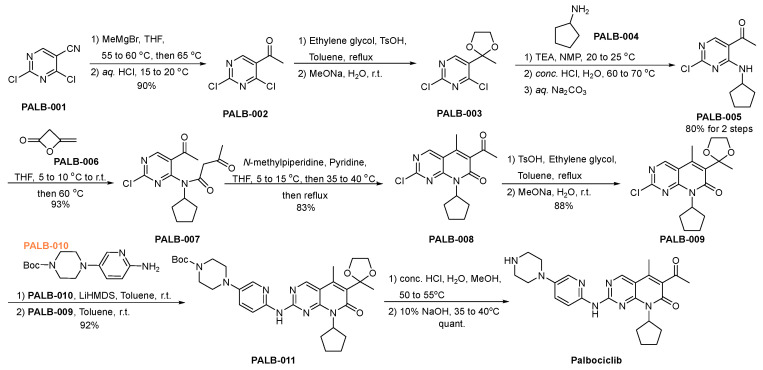
Synthetic route of Palbociclib.

The preparation of Palbociclib begins with the reaction between 2,4-dichloropyrimidine-5-carbonitrile (**PALB-001**) and methylmagnesium bromide, yielding ketone **PALB-002** (Scheme 1) [19]. Subsequently, the carbonyl group of **PALB-002** is protected using ethylene glycol, affording ketal **PALB-003**. Next, amination with cyclopentanamine (**PALB-004**) produces the secondary amine **PALB-005**. This intermediate undergoes acylation with 4-methyleneoxetan-2-one **(PALB-006**), yielding amide **PALB-007**. **PALB-007** is then dehydrated to form the cyclization product **PALB-008**, which is subsequently protected using ethylene glycol to create ketal **PALB-009**. A nucleophilic aromatic substitution (S*_N_*Ar) reaction of **PALB-009** with aminopyridine (**PALB-010**) yields **PALB-011**, which undergoes hydrolysis to produce Palbociclib.

### 3.2. Ribociclib

Ribociclib Succinate, marketed under the trade name Kisqali, was granted approval by the FDA in the United States on 13 March 2017, and it was subsequently introduced to the Chinese and European markets for use in combination with aromatase inhibitors as the first-line endocrine therapy for postmenopausal women diagnosed with hormone receptor-positive (HR+), HER2- advanced, or metastatic breast cancer [9]. Ribociclib is an extremely selective inhibitor of CDK4/6 (IC_50_ = 10 nM CDK4, 39 nM for CDK6) [20]. By obstructing the activity of CDK4/6, Ribociclib effectively hampers the transition of cancer cells from the G1 phase to the S phase, thereby impeding their entry into the S phase. The inhibition of CDK4/6 prevents cell division and proliferation, ultimately restraining the growth of cancer cells [21]. Frequent adverse effects of Ribociclib may include a decrease in neutrophil count, potential liver damage, tiredness, feeling sick, and prolongation of the QT interval [9]. It is important to note that Ribociclib should not be used in patients with congenital long QT syndrome or those who have a known hypersensitivity to the medication or any of its components.

**Scheme 2:**
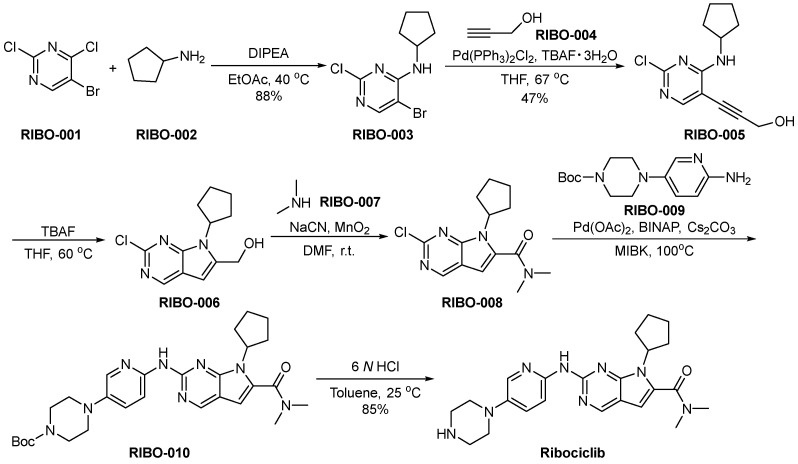
Synthetic route of Ribociclib.

The commercially available 5-bromo-2,4-dichloropyrimidine (**RIBO-001**) undergoes selective substitution upon reaction with cyclopentanamine (**RIBO-002**), leading to the formation of **RIBO-003** (Scheme 2) [22]. The combination of **RIBO-003** and propargyl alcohol (**RIBO-004**) yields **RIBO-005**. Subsequently, the intramolecular cyclization of **RIBO-005** is achieved by employing tetrabutylammonium fluoride (TBAF) in tetrahydrofuran (THF), affording pyrrolo[2,3-*d*]pyrimidine **RIBO-006**. The next process involves substituting with dimethylamine (**RIBO-007**) and subsequently oxidizing to form amide **RIBO-008**. The subsequent step entails coupling **RIBO-008** with amine **RIBO-009**, followed by deprotecting *t*-butyloxy carbonyl (Boc) to ultimately obtain Ribociclib.

### 3.3. Abemaciclib

Abemaciclib, developed by Eli Lilly and marketed under the trade name Verzenio, was approved by the FDA in 2017 in the United States [10]. This drug was subsequently introduced to the Chinese, Japanese, and European markets. It is indicated for the treatment of postmenopausal women diagnosed with HR+, HER2- advanced, or metastatic breast cancer, either in combination with Fulvestrant or as a monotherapy, whose disease has progressed following endocrine therapy [23]. Abemaciclib is a potent inhibitor of CDK4 and CDK6 (IC_50_ = 2 nM for CDK4, IC_50_ = 10 nM for CDK6) [24]. The results of in vitro experiments demonstrate that prolonged exposure to Abemaciclib effectively inhibits the phosphorylation of Rb, thereby impeding cell cycle progression from the G1 phase to the S phase, consequently leading to cellular senescence and apoptosis [25]. The daily administration of Abemaciclib, either alone or in combination with anti-estrogen drugs, led to a decrease in tumor volume [26]. Common side effects include gastrointestinal disturbances like diarrhea and abdominal pain, as well as fatigue, neutropenia, and nausea [10]. Abemaciclib should not be used in patients with severe liver problems or a known hypersensitivity to the drug or any of its ingredients.

**Scheme 3:**
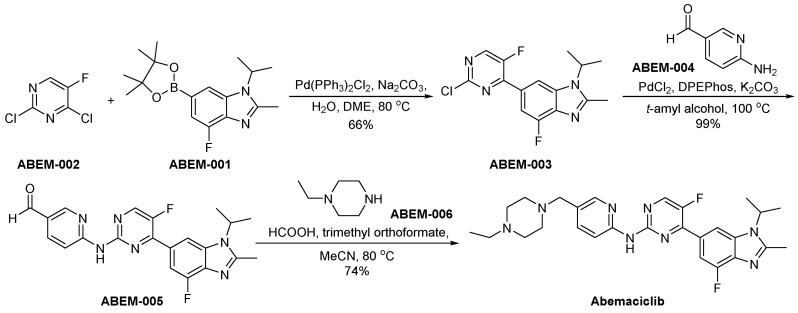
Synthetic route of Abemaciclib.

The process of producing Abemaciclib begins with the Suzuki coupling reaction, where the chloride located at position 4 in dichloro-pyrimidine **ABEM-002** is combined with boronic ester **ABEM-001** (Scheme 3) [27]. This reaction yields biaryl compound **ABEM-003**, which is subsequently subjected to Buchwald–Hartwig amination with 6-aminonicotinaldehyde (**ABEM-004**) to produce aldehyde **ABEM-005**. Finally, Abemaciclib is obtained by the reaction of **ABEM-005** with *N*-ethylpiperazine (**ABEM-006**) through reductive amination utilizing Leuckart–Wallach conditions.

### 3.4. Trilaciclib

Developed by G1 therapeutics and marketed under the trade name Cosela, Trilaciclib was approved by the FDA in the United States on 12 February 2021, which was also commercialized in China. The short-acting nature of this compound, when combined with cancer chemotherapy, offers myeloprotection and holds the potential to demonstrate antitumor efficacy [11]. Trilaciclib’s IC_50_s is 1 nM for CDK4 and 4 nM for CDK6 [28]. Trilaciclib mitigates the detrimental effects of chemotherapy on hematopoietic stem and progenitor cells by impeding the phosphorylation mechanism of retinoblastoma protein (Rb), resulting in a decrease in myelosuppression. Animal experimentation has provided evidence that Trilaciclib effectively alleviates the burden on bone marrow hematopoietic progenitor cells by temporarily obstructing the G1 phase, thereby preventing the depletion of bone marrow hematopoietic stem cells. This mechanism proves advantageous in safeguarding hematopoietic stem cells, progenitor cells, and the immune system during chemotherapy [29]. Adverse effects that commonly occur include nausea, fatigue, headache, and bone marrow suppression, resulting in decreased levels of neutrophils and white blood cells [11]. Trilaciclib is contraindicated in patients who have a known hypersensitivity to the drug or any of its components.

**Scheme 4:**
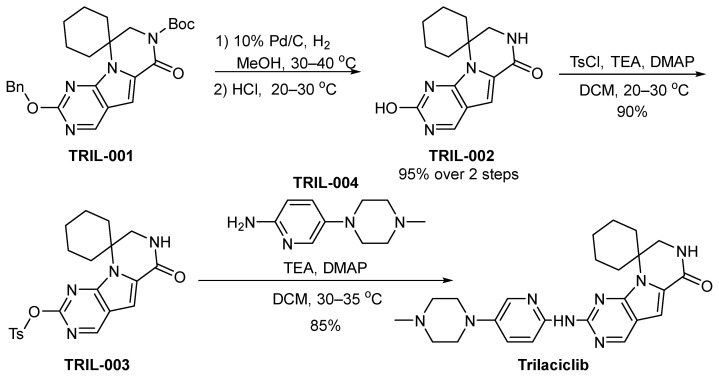
Synthetic route of Trilaciclib.

The treatment of **TRIL-001** with H_2_, catalyzed by Pd/C, removes the benzyl (Bn) group, followed by the removal of boc to obtain alcohol **TRIL-002** (Scheme 4) [30]. Subsequently, substitution with tosyl chloride (TsCl) leads to the formation of **TRIL-003**, which further undergoes substitution with amine **TRIL-004** to give Trilaciclib.

### 3.5. Dalpiciclib Isethionate

Dalpiciclib Isethionate is an innovative drug created by Jiangsu Hengrui Medicine Co., Ltd. (Lianyungang, China). In December 2021, the National Medical Products Administration (NMPA) in China approved the drug to be used in combination with Fluvisquine to treat patients with HR+ and HER2- recurrent or metastatic breast cancer who have experienced disease progression after previous endocrine therapy [31]. Dalpiciclib is a potent and specific inhibitor of CDK4 and 6, which can be taken orally (IC_50_ = 12.4 nM for CDK4, IC_50_ = 9.9 nM for CDK6) [32]. The potential of decreasing Rb phosphorylation levels in response to the CDK4 and CDK6 signaling pathways can lead to cell cycle arrest in the G1 phase, thereby inhibiting tumor cell proliferation. The compound effectively decreases Rb phosphorylation, a crucial step in the CDK4 and CDK6 signaling pathways. This results in G1 phase cell cycle arrest, which hinders the growth of cancerous cells by preventing their proliferation [33]. Common adverse effects associated with dalpiciclib include neutropenia, leukopenia, fatigue, nausea, and diarrhea [31]. Patients with severe hepatic impairment or individuals with a known hypersensitivity to the drug or any of its components should refrain from using Dalpiciclib.

**Scheme 5:**
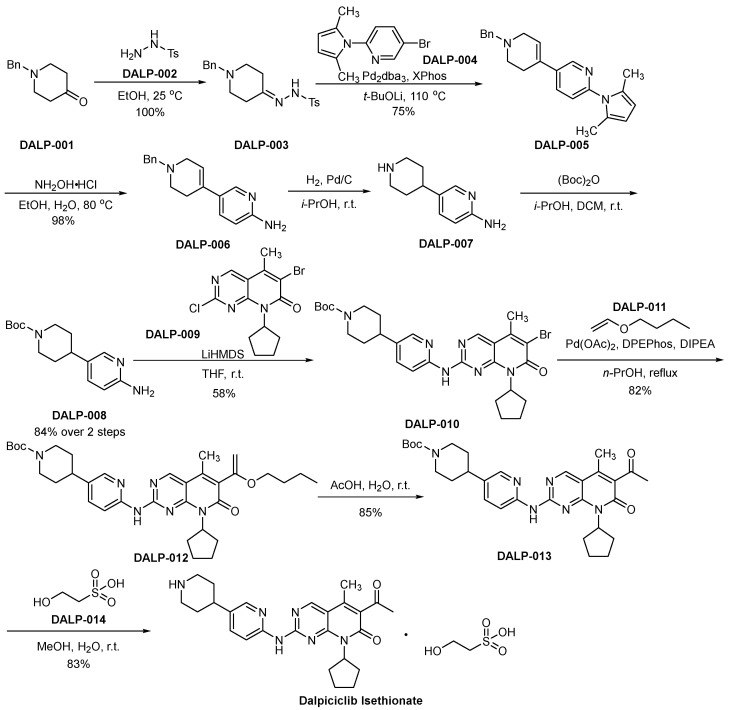
Synthetic route of Dalpiciclib Isethionate.

The synthesis begins with the condensation reaction between piperidinone **DALP-001** and hydrazine **DALP-002** in alcohol, leading to the formation of hydrazone **DALP-003** (Scheme 5) [34]. Subsequently, **DALP-003** is coupled with bromopyridine **DALP-004** to yield **DALP-005**, which undergoes deprotection using hydroxylamine to generate amine **DALP-006**. The reduction in the cyclohexene moiety is achieved through hydrogenation with H_2_, leading to the formation of the crucial intermediate **DALP-007**. The reaction of **DALP-007** with (Boc)_2_O affords the *N*-Boc protected compound, namely *N*-Boc-**DALP-008**. The substitution of a chlorine atom in **DALP-009** gives rise to the product **DALP-010**, which subsequently reacts with 1-(vinyloxy)butane (**DALP-011**) to produce **DALP-012**. The cleavage of the ether bond within **DALP-012** using AcOH and subsequent tautomerism results in ketone derivative **DALP-013**. The deprotection and salt formation reactions are carried out in one step through the treatment of **DALP-013** with isethionic acid in methanol and water to give Dalpiciclib Isethionate.

### 3.6. Dinaciclib

Dinaciclib is currently involved in a Phase I clinical trial for the treatment of acute myeloid leukemia (AML) [35]. Dinaciclib is a highly effective CDK inhibitor, exhibiting IC_50_ values of 1 nM for CDK2, 1 nM for CDK5, 3 nM for CDK1, and 4 nM for CDK9 [36]. Dinaciclib effectively inhibits DNA replication and the uptake of thymidine (dThd) into DNA (IC_50_ = 4 nM) in A2780 cells [36]. The complete inhibition of Rb phosphorylation is achieved by Dinaciclib at 6.25 nM, resulting in cell cycle arrest and apoptosis in cancer cells. This effect is supported by the presence of cleavage products of p85 poly ADP-ribose polymerase (PARP). Dinaciclib has demonstrated efficacy against various human tumor cell lines [36].

**Scheme 6:**
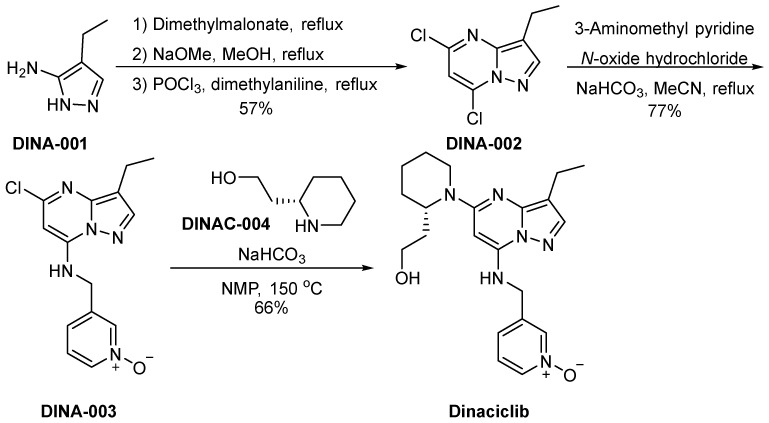
Synthetic route of Dinaciclib.

The corresponding 3-ethyl derivative **DINA-002** is synthesized by cyclization between amino pyrazole **DINA-001** and dimethylmalonate, followed by chlorination (Scheme 6) [37]. The treatment of **DINA-002** with 3-(aminomethyl)pyridine *N*-oxide monohydrochloride yields **DINA-003**. Subsequently, the reaction of **DINA-003** with (*S*)-2-(piperidin-2-yl)ethan-1-ol (**DINA-004**) leads to the formation of Dinaciclib.

### 3.7. Ebvaciclib

Ebvaciclib, developed by Pfizer Inc., not only demonstrates potent pharmacological activity and inhibits tumor growth in multiple cancer models by targeting CDK2/4/6, but it also has the potential to enhance the anti-tumor immune response [38]. Based on the clinical characteristics of resistance to CDK4/6 inhibitors/anti-hormone combination therapy in in vitro and in vivo studies, the activation of MYC (MYC proto-oncogene, bHLH transcription factor) and CDK2 serves as a compensatory resistance mechanism [39]. Ebvaciclib, the first targeted therapeutic drug to undergo clinical trials for cancer patients, is a highly effective and orally accessible small-molecule inhibitor of CDK2/4/6. The inhibition of these kinases leads to cell cycle arrest, the induction of apoptosis, and the inhibition of tumor cell proliferation. Despite undergoing Phase I clinical trials, it is likely to effectively address the treatment challenges in patients who have developed resistance to CDK4/6 inhibitors [40].

**Scheme 7:**
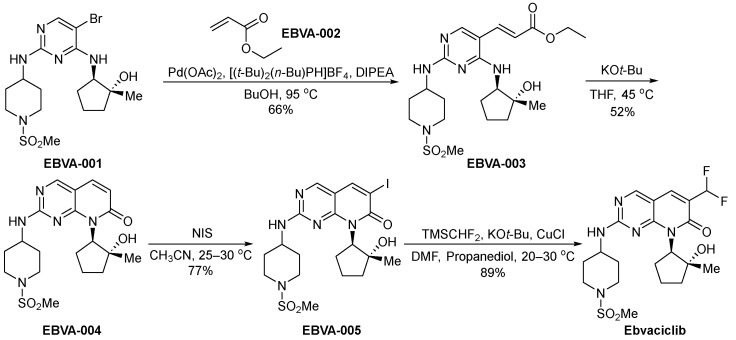
Synthetic route of Ebvaciclib.

The Heck coupling reaction between **EBVA-001** and ethyl acrylate (**EBVA-002**) affords the ester **EBVA-003**, which is subsequently subjected to intramolecular ammonolysis to yield pyrimidinone **EBVA-004** (Scheme 7) [41]. The iodization of **EBVA-004** affords **EBVA-005**, which undergoes alkylation with (difluoromethyl)trimethylsilane (TMSCHF2), resulting in the formation of Ebvaciclib.

### 3.8. Monepantel

Monepantel is a veterinary drug that has been authorized for the treatment of nematode infections in livestock. A flow cytometry analysis of cells treated with Monepantel has shown that it induces cell cycle arrest at the G1 phase, suggesting its potential as an anticancer agent [42]. The expressions of cyclins D1 and A were downregulated in Monepantel-treated cells, whereas the expression of cyclin E2 was upregulated. Consistent with G1 phase arrest, the levels of CDK 2 and 4 were reduced in the cells, while the expression of the CDK inhibitor p27(kip) was elevated [43]. Currently, this drug is undergoing Phase I clinical trials in Australia, focusing on its potential for treating motor neuron disease.

**Scheme 8:**
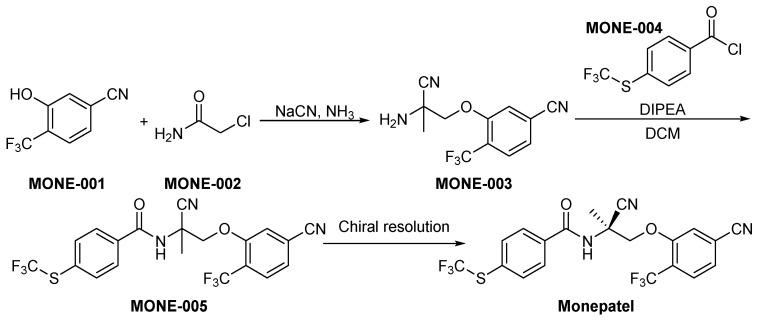
Synthetic route of Monepantel.

The reaction between 3-hydroxy-4-(trifluoromethyl)benzonitrile (**MONE-001**) and 2-chloroacetamide (**MONE-002**) leads to the formation of the ester **MONE-003** (Scheme 8) [44]. Subsequent condensation with 4-((trifluoromethyl)thio)benzoyl chloride (**MONE-004**) results in the synthesis of the amide **MONE-005**. Racemic **MONE-005** is then resolved to obtain its corresponding chiral form, Monepantel.

### 3.9. TP-1287

TP-1287 is an orally administered phosphate salt of Alvocidib, a CDK9 inhibitor, which is currently being investigated as an experimental prodrug [45]. It is metabolized to Alvocidib through enzymatic cleavage [46]. Alvocidib specifically targets the ATP binding site in CDK9, leading to the inhibition of its phosphorylation activity. This interference ultimately disrupts the productive transcription process and leads to a decrease in messenger RNA (mRNA) levels for various genes, such as c-MYC and myeloid cell leukemia-1 (MCL-1). Furthermore, the apoptosis of different tumor cells is induced by the downregulation of c-MYC and MCL-1 transcription [47]. Phase I clinical trials are currently being conducted on TP-1287 to evaluate its effectiveness in the treatment of ewing sarcoma, liposarcoma, and synovial sarcoma [48,49,50].

**Scheme 9:**
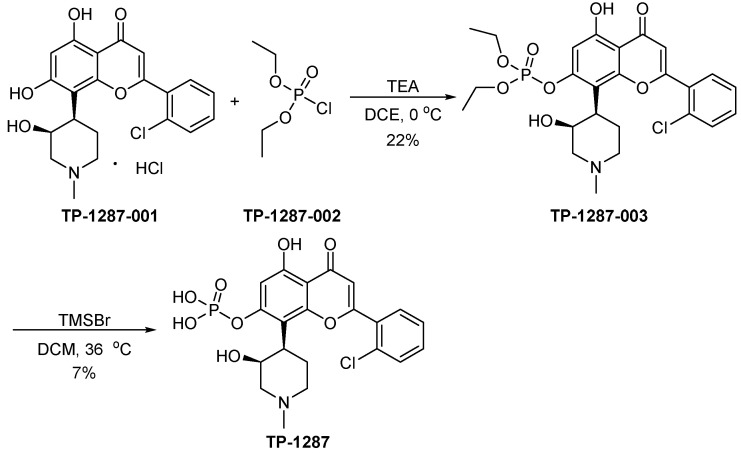
Synthetic route of TP-1287.

The phenol **TP-1287-001** solution in dichloroethane (DCE) is combined with diethylchlorophosphate (**TP-1287-002**) and triethylamine (TEA) at 0 °C to yield **TP-1287-003** with a 22% conversion rate (Scheme 9). **TP-1287** is obtained in a 7% yield through the treatment of bromotrimethylsilane (TMSBr) in dichloromethane (DCM) [51].

### 3.10. Enitociclib

Enitociclib, a pharmaceutical product created by Bayer AG, is presently undergoing Phase II clinical trials to assess its efficacy in managing double-hit diffuse large B-cell lymphoma (DH-DLBCL). Enitociclib, a potent CDK9 inhibitor (IC_50_ = 3 nM), has demonstrated favorable outcomes in terms of tolerability and clinical effectiveness. Notably, when administered intravenously at a dosage of 30 mg once a week, it has led to complete metabolic remissions in two out of seven patients with DH-DLBCL [52]. DH-DLBCL is a highly aggressive type of B-cell non-Hodgkin lymphoma (NHL) that often shows resistance to treatment [53]. It is characterized by genetic modifications in MYC and B-cell lymphoma-2 (BCL-2). CDK9 plays a crucial role in regulating transcriptional elongation and activating transcription factors like MYC. This implies that targeting CDK9 could be considered as a potential therapeutic approach for treating MYC-positive lymphomas [54].

**Scheme 10:**
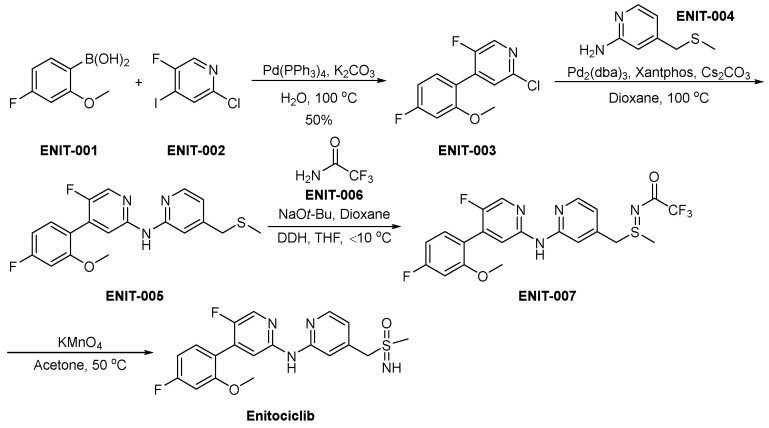
Synthetic route of Enitociclib.

The coupling reaction between boronic acid **ENIT-001** and iodopyridine **ENIT-002** leads to the formation of **ENIT-003** through the Suzuki coupling reaction (Scheme 10) [52]. Subsequently, the cross-coupling reaction between **ENIT-003** and 4-((methylthio)methyl)pyridin-2-amine (**ENIT-004**), known as Buchwald–Hartwig cross-coupling, results in the production of **ENIT-005**. The thioether **ENIT-005** reacts with 2,2,2-trifluoroacetamide (**ENIT-006**), followed by oxidation with KMnO_4_, yielding Enitociclib.

### 3.11. FIT-039

The efficacy of FIT-039 in treating uterine cervical dysplasia and warts is currently being assessed through two distinct Phase II clinical studies. FIT-039, an orally active CDK9 inhibitor (IC_50_ = 5.8 μM), selectively targets and competes with ATP. Research has demonstrated its potential not only for treating cervical tumors, but also for treating hepatitis B and human immunodeficiency virus (HIV)/acquired immune deficiency syndrome (AIDS). By inhibiting mRNA transcription, FIT-039 effectively suppresses replication in a wide range of DNA viruses. In cultured cells, it has been found to significantly hinder the replication of various viruses including herpes simplex virus 1 (HSV-1), HSV-2, human cytomegalovirus, and human adenovirus. Furthermore, topical application of FIT-039 ointment successfully suppressed the formation of skin lesions in a mouse model infected with HSV-1 [55].

**Scheme 11:**
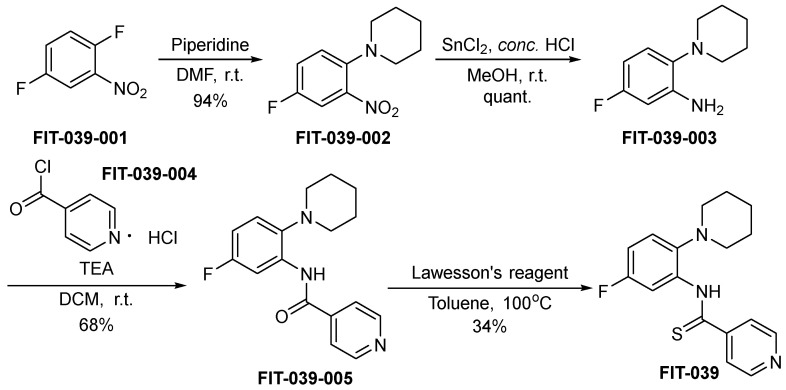
Synthetic route of FIT-039.

Commercially available 1,4-difluoro-2-nitrobenzene (**FIT-039-001**) undergoes substitution with piperidine to give **FIT-039-002** (Scheme 11) [56]. The resulting nitro group of **FIT-039-002** is subsequently reduced to form benzylamine **FIT-039-003**. The treatment of **FIT-039-003** with acyl chloride **FIT-039-004** leads to the formation of amide compound **FIT-039-005**. Finally, the synthesis of **FIT-039** is accomplished by treating Lawesson’s reagent via thiocarbonylation.

### 3.12. Seliciclib

Seliciclib is currently undergoing Phase II clinical trials for the treatment of cystic fibrosis (CF), rheumatoid arthritis (RA), Cushing syndrome (CS), and nasopharyngeal carcinoma (NPC), which has demonstrated a high degree of oral bioavailability [57,58,59,60]. Seliciclib is an inhibitor that selectively targets CDKs (IC_50_ = 0.2 μM for CDK5, 0.7 μM for CDK2) [61]. The main way it works is by inhibiting transcription, which leads to the targeted decrease in the production of rapidly cycling mRNA transcripts, such as MCL-1 and cyclin D1. The compound demonstrates autonomous antitumor properties and enhances the effectiveness of various cytotoxic and targeted drugs [62].

**Scheme 12:**
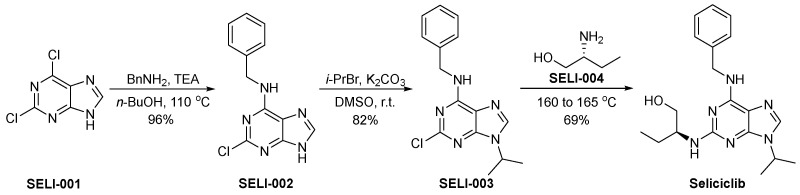
Synthetic route of Seliciclib.

The synthesis of Seliciclib is outlined in Scheme 12, commencing with the substitution reaction between the 6-chloro group of 2,6-dichloropurine (**SELI-001**) and benzylamine under basic conditions to yield **SELI-002** [63]. Subsequently, **SELI-002** undergoes a reaction with isopropyl bromide to afford **SELI-003**. The resulting chloropurine derivative, **SELI-003**, is then subjected to (*R*)-2-amino-1-butanol for the production of Seliciclib.

### 3.13. Zotiraciclib

Zotiraciclib, an orally available CDK2inhibitor, possesses the capability to penetrate the blood–brain barrier and efficiently degrade cancer-associated proteins MCL-1 and MYC [64]. Zotiraciclib effectively inhibits CDK2 (IC_50_ = 13 nM), janus kinase 2 (JAK2) (IC_50_ = 73 nM), and fms-like tyrosine kinase 3 (FLT3) (IC_50_ = 56 nM) [65]. Zotiraciclib is currently undergoing evaluation in two distinct Phase II clinical trials to determine its ability to treat brain cancer and neoplasms. The molecular structure of Zotiraciclib is distinguished by a large ring configuration, rendering it a unique compound. The conformation of the large ring facilitates the presentation of key pharmacophores when binding to proteins, the high activity of which can be attributed to this interaction.

**Scheme 13:**
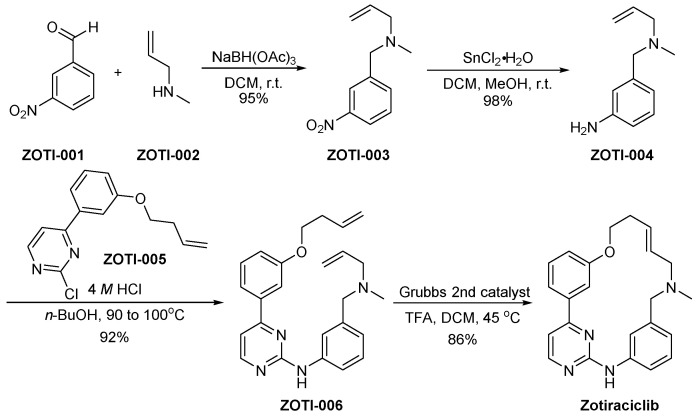
Synthetic route of Zotiraciclib.

The reductive amination of benzaldehyde **ZOTI-001** with allylamine **ZOTI-002** leads to the formation of **ZOTI-003** in a 95% yield (Scheme 13) [65]. Subsequently, the nitro group of **ZOTI-003** is reduced under SnCl_2_·H_2_O, giving **ZOTI-004**. The coupling reaction between **ZOTI-004** and chloropyrimidine **ZOTI-005** takes place in hot butanol, yielding **ZOTI-006**. Finally, the macrocyclization of **ZOTI-006** using Grubb’s second-generation catalyst affords Zotiraciclib.

### 3.14. BPI-16350

Developed by Beida Pharmaceuticals, BPI-16350 is being evaluated in Phase III clinical trials. BPI-16350 has been developed with a specific focus on CDK4/6 inhibition, making it suitable for both standalone use and combination therapy with hormone treatment in the management of HR+/HER2- patients diagnosed with advanced or metastatic breast cancer [66]. The compound can also function as a primary or secondary treatment modality, either as monotherapy or in combination with other therapeutic approaches, for cancers with Rb+ expression. Preclinical evidence demonstrates the consistent and robust biological activity of BPI-16350 in both in vitro and in vivo animal models. It effectively inhibits the proliferation of diverse types of solid tumor cells and exhibits potent anti-tumor effects when administered alone or concurrently with other medications across various solid tumor models. Moreover, it possesses excellent physicochemical characteristics and pharmacokinetic properties [67].

**Scheme 14:**
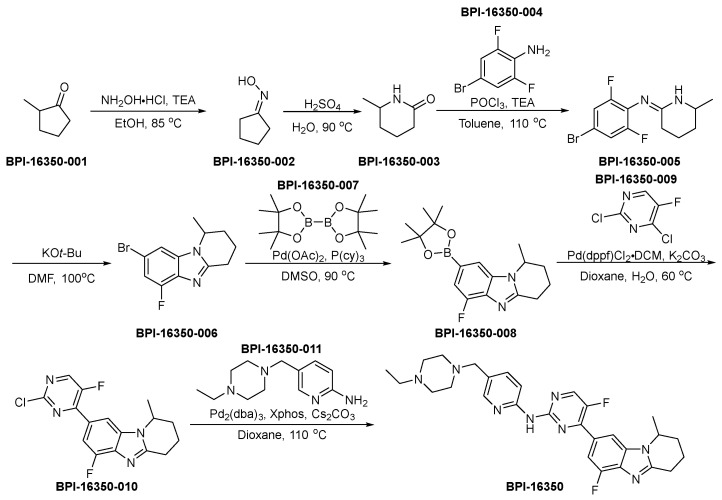
Synthetic route of BPI-16350.

The commercially available 2-methylcyclopentanone (**BPI-16350-001**) is condensed with hydroxylamine to yield oxime **BPI-16350-002** (Scheme 14) [68]. The Beckmann rearrangement of **BPI-16350-002** affords lactam **BPI-16350-003**, which is then treated with 4-bromo-2,6-difluoroaniline (**BPI-16350-004**). The resulting imine **BPI-16350-005** is subjected to intramolecular cyclization, yielding **BPI-16350-006**. The hydrocarbon bromide **BPI-16350-006** is then converted to boron compound **BPI-16350-008** by the Miyaura borylation reaction. After Suzuki coupling with 2,4-dichloro-5-fluoropyrimidine (**BPI-16350-009**) and further coupling with amine **BPI-16350-011**, BPI-16350 is obtained.

### 3.15. Rigosertib Sodium

Rigosertib Sodium, an experimental drug being developed by Onconova Therapeutics, Inc., is currently undergoing Phase III clinical trials. Its primary focus lies in the treatment of refractory anemia with excess blasts and chronic myelomonocytic leukemia (CML), showcasing its potential as a promising antineoplastic agent [69]. When administered, Rigosertib specifically targets and attaches to the Ras-binding domain (RBD) that is present in various Ras effector proteins, such as Raf kinase and phosphatidylinositol 3-kinase (PI3K). This hinders the binding of Ras to its targets and suppresses the Ras-mediated signaling pathways, such as the Ras/Raf/Erk, Ras/CRAF/polo-like kinase1 (Plk1), and Ras/PI3K/Akt signaling pathways [70]. Rigosertib has the ability to cause cell cycle arrest and apoptosis, leading to a decrease in cell proliferation in various types of tumor cells. Additionally, it acts as a CDK1 inhibitor (IC_50_ = 260 nM) [71].

**Scheme 15:**
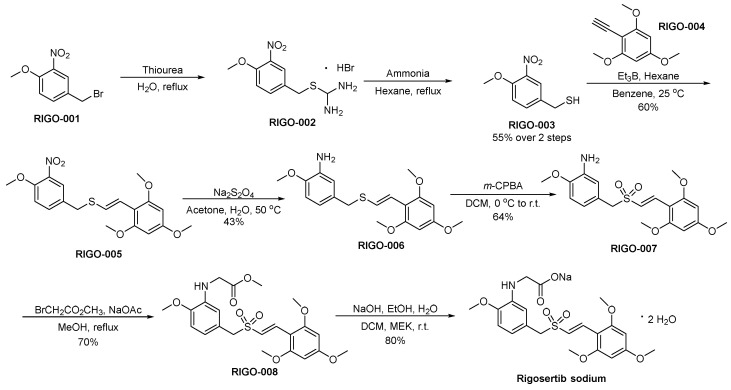
Synthetic route of Rigosertib Sodium.

Rigosertib Sodium is synthesized from 4-(bromomethyl)-1-methoxy-2-nitrobenzene (**RIGO-001**), as outlined in Scheme 15 [72,73]. When thiourea is treated with **RIGO-001**, it forms isothiouronium salt **RIGO-002**, which is further reduced with ammonia to produce thiol **RIGO-003**. Through the reaction of **RIGO-003** and phenylacetylene **RIGO-004** in the presence of triethylborane-hexane (Et3B), **RIGO-005** is afforded. The conversion of nitro sulfide **RIGO-005** into amino sulfide **RIGO-006** can be achieved, followed by oxidation using m-chloroperoxybenzoic acid (*m*-CPBA) to produce sulfone **RIGO-007**. Subsequently, when methyl 2-bromoacetate reacts with **RIGO-007** in methanol, ester formation occurs, leading to the generation of compound **RIGO -008**. The hydrolysis of this compound through treatment with NaOH in a mixture containing aqueous ethanol and DCM, followed by washing with methyl ethyl ketone (MEK), ultimately yields a crystalline water-containing form of Rigosertib Sodium.

### 3.16. Birociclib

Xuanzhu Biopharmaceutical Co., Ltd. (Shijiazhuang, China) has submitted a new drug application for its new independently developed Class 1 drug, Birociclib (CDK4/6 inhibitor), to the NMPA for review and approval. Birociclib monotherapy is used to treat adult patients with locally advanced or metastatic HR+ or HER2- breast cancer who have previously received two or more endocrine therapies and one chemotherapy and have experienced disease progression [74]. Preclinical studies have shown that Birociclib possesses distinctive pharmacokinetic properties and demonstrates remarkable ability to traverse the blood–brain barrier. It is anticipated to exhibit favorable therapeutic outcomes in individuals afflicted with brain metastasis of breast cancer as well as primary brain tumors [75].

**Scheme 16:**
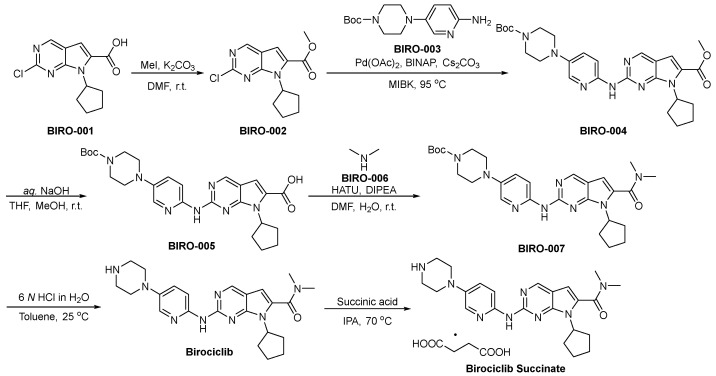
Synthetic route of Birociclib Succinate.

The substitution of carboxylic acid **BIRO-001** with methyl iodide in the presence of K_2_CO_3_ furnishes ester **BIRO-002** (Scheme 16) [76]. The coupling of **BIRO-002** and amine **BIRO-003** yields **BIRO-004**. The hydrolysis of **BIRO-004** affords carboxylic acid **BIRO-005**. The treatment of **BIRO-005** with dimethylamine (**BIRO-006**) provides amide **BIRO-007**, followed by deprotection of the boc group to provide Birociclib, which is then converted to Birociclib Succinate in the presence of succinic acid.

### 3.17. Lerociclib

Lerociclib, developed by G1 Therapeutics, Inc., which is intended to treat HR+/HER2- breast cancer, is currently undergoing the process of seeking approval for market release in China. Lerociclib is a highly effective and specific inhibitor of CDK4/6 (IC_50_ = 1 nM for CDK4, IC_50_ = 2 nM for CDK6) [77]. Research data suggest that the combination of Lerociclib with Fulvestrant significantly prolongs the PFS time in patients and improves the response rate compared to the combination of Fulvestrant with a placebo [78]. In terms of toxicity, the occurrence of severe neutropenia (grade 4) was minimal (5.1%), and there were no instances of febrile neutropenia or significant diarrhea (grade 3/4). The frequency of abnormal liver function is comparable between the two groups, and both groups exhibited a low incidence of rash at approximately 4% [79].

**Scheme 17:**
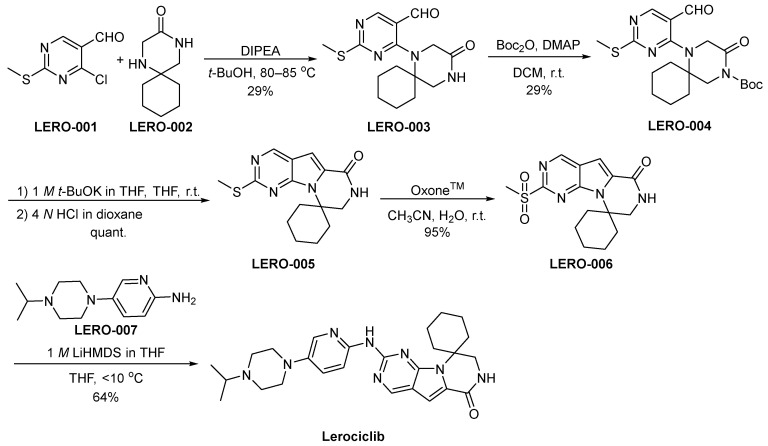
Synthetic route of Lerociclib.

The synthesis of Lerociclib commences with nucleophilic substitution between **LERO-001** and spirolactam (**LERO-002)**, facilitated by diisopropylethylamine (DIPEA), yielding **LERO-003** (Scheme 17) [80]. Subsequently, **LERO-003** undergoes a reaction with Boc_2_O to generate *N*-Boc protected **LERO-004**. The intramolecular condensation of **LERO-004** is achieved through treatment with *t*-BuOK, followed by deprotection, resulting in the formation of **LERO-005**. The oxidation of **LERO-005** using oxone affords **LERO-006**. Finally, the substitution of an amine group onto **LERO-006** yields Lerociclib.

## 4. Discussion and Conclusions

One of the primary obstacles encountered in the development of small-molecule inhibitors for CDKs is attaining a high level of selectivity. CDKs are part of a vast family of kinases, many of which possess similar structures, particularly within the ATP-binding pocket. This resemblance presents a significant challenge when designing inhibitors that can specifically target distinct isoforms of CDKs. It is crucial to achieve isoform specificity to minimize any potential off-target effects and toxicity risks. Synthetic chemists bear the responsibility for creating compounds that are capable of distinguishing between closely related CDK isoforms. This necessitates an extensive comprehension of structural variations and their functional implications when targeting specific isoforms. By employing selective targeting, inadvertent interactions with other kinases can be diminished, thereby reducing side effects and enhancing therapeutic outcomes. For instance, Palbociclib was formulated as an exceedingly precise inhibitor for CDK4/6 to exclusively target these kinases in hormone receptor-positive breast cancer, where their dysregulation significantly contributes to disease progression.

Another significant obstacle is the issue of drug resistance. The effectiveness of CDK inhibitors against cancer cells can be compromised by various mechanisms, such as mutations occurring in the targeted kinase or the activation of alternative signaling pathways. For instance, mutations within the ATP-binding domain of CDKs can impede inhibitor binding and diminish drug efficacy. Additionally, cancer cells may activate compensatory signaling pathways that bypass the need for CDK activity, thereby reducing the potency of CDK inhibitors. To address these resistance mechanisms, it is imperative to develop synthetic strategies that design inhibitors capable of overcoming or circumventing them. This could involve creating advanced inhibitors targeting multiple CDK isoforms or utilizing combination therapies simultaneously targeting both CDKs and other signaling pathways, like PI3K or mTOR. By targeting multiple CDKs or combining CDK inhibitors with parallel pathway inhibitors, like PI3K or mTOR, it becomes possible to potentially overcome resistance and achieve more enduring responses.

The advent of covalent inhibitors offers an auspicious pathway for investigation. These inhibitors forge enduring connections with their designated targets, leading to persistent inhibition. Through the precise targeting of distinctive cysteine residues adjacent to CDKs’ active sites, covalent inhibitors can attain heightened selectivity and potency. This strategy holds promise in mitigating concerns related to selectivity and resistance as the establishment of a covalent bond diminishes the probability of resistance mutations while augmenting inhibitor efficacy. The irreversibility of binding guarantees uninterrupted suppression of CDKs, rendering it especially advantageous when confronting cancer cells marked by accelerated proliferation rates.

Another potential lies in improving the pharmacokinetic properties of CDK inhibitors, such as their solubility, stability, and bioavailability. By optimizing these characteristics, synthetic chemists have the opportunity to enhance the effectiveness and safety profiles of CDK inhibitors. Advanced techniques in medicinal chemistry, including structure-based drug design and high-throughput screening, can be utilized to optimize these parameters. This optimization process can lead to the development of more efficient and patient-friendly therapies. For instance, modifications that boost the metabolic stability of CDK inhibitors may result in enhanced oral bioavailability and longer half-lives, thus increasing convenience for patients.

In the clinical setting, there is promising evidence for the effectiveness of CDK inhibitors in treating various cancers, particularly hormone receptor-positive breast cancer. Ongoing studies are investigating their potential when used alongside other therapies. The combination of CDK inhibitors with targeted treatments or chemotherapy has been shown to enhance their efficacy and overcome resistance mechanisms. For instance, the combination of CDK4/6 inhibitors with endocrine therapy has demonstrated significant improvements in outcomes for patients with breast cancer. This approach can be extended to different types of cancer, potentially expanding the therapeutic applications of CDK inhibitors. Additionally, current research aims to identify drug combinations that work synergistically to provide maximum therapeutic benefits while minimizing toxicity.

The significance of targeting specific CDK inhibitors, such as those that inhibit CDK4/6, stems from their crucial role in regulating the cell cycle, particularly in cancers where these kinases are dysregulated. By specifically focusing on CDK4/6 inhibition, these inhibitors have the ability to impede cancer cell proliferation, resulting in tumor reduction and extended patient survival. The selection of molecules within this category, including Palbociclib, Ribociclib, and Abemaciclib, is based on their capacity to selectively hinder CDK4/6 activity, which tends to be excessively active in hormone receptor-positive breast cancer. These inhibitors have demonstrated significant clinical advantages by enhancing progression-free survival and overall survival rates during clinical trials, underscoring their potential for therapeutic use.

In summary, the control of the cancer cell cycle is heavily dependent on CDKs. The exploration of CDKs as potential targets for successful cancer treatment has gained considerable interest. Considerable endeavors have been made in the realm of pharmaceutical investigation concerning the advancement of small-molecule inhibitors that selectively focus on CDKs. However, the development of small-molecule inhibitors of CDKs presents both challenges and opportunities in synthetic approaches and clinical applications. Achieving isoform selectivity and overcoming drug resistance are major challenges that synthetic chemists need to address. However, the improvement of covalent inhibitors and the optimization of pharmacokinetics play a crucial role in enhancing the specificity and effectiveness of CDK inhibitors. Covalent inhibitors, through the formation of irreversible bonds, offer long-lasting inhibition to their targets, which proves particularly beneficial in treating aggressive forms of cancer. Pharmacokinetic optimization ensures that these inhibitors are well tolerated; possess favorable absorption, distribution, metabolism, and excretion (ADME) characteristics; and can be conveniently administered to enhance patient compliance and overall outcomes. In clinical settings, the potential of CDK inhibitors in treating various cancers has been demonstrated, and ongoing studies are investigating their effectiveness when used in combination therapies. This review presents a comprehensive examination of the synthetic methods of 17 representative small-molecule CDK inhibitors that have received regulatory approval or are currently undergoing evaluation through clinical trials, as well as their mechanisms of action in clinical settings, thereby potentially expediting the development of novel CDK inhibitors for cancer treatment.

## Data Availability

Data are contained within the article.
